# Supplementary material for: Measuring Strong, Skillful, Good and Transpersonal Will: The development of the Multidimensional Will Scale
Source: PLoS One. 2024 Jul 11;19(7):e0305477. doi: 10.1371/journal.pone.0305477 (PMC11239019; doi:10.1371/journal.pone.0305477)
Supplement: S1 Table — B. Factor Loadings, inter-item correlations and internal consistency of the 30-item solution of the Multidimensional Will Scale (MWS). C. Factor Loadings, inter-item correlations, and internal consistency of the 23-item Multidimensional Will Scale (MWS). (DOCX) [file pone.0305477.s001.docx]

**Supplementary Material**

**S1 Table A.** *Descriptive statistics for 38-item Multidimensional Will Scale (MWS).*

| **ITEM** | ***M*** | ***SD*** | ***Sk*** | ***Ku*** | ***Min*** | ***Max*** |
| --- | --- | --- | --- | --- | --- | --- |
| **IT 1** | 3.50 | .79 | -.47 | .29 | 1 | 5 |
| **IT 2** | 4.13 | .76 | -.74 | .60 | 1 | 5 |
| **IT 3** | 3.92 | .76 | -.50 | .44 | 1 | 5 |
| **IT 4** | 2.32 | 1.13 | .57 | -.53 | 1 | 5 |
| **IT 5** | 4.07 | .80 | -.55 | -.12 | 1 | 5 |
| **IT 6** | 3.80 | .92 | -.38 | -.36 | 1 | 5 |
| **IT 7** | 1.91 | 1.11 | 1.12 | .41 | 1 | 5 |
| **IT 8** | 3.85 | .82 | -.33 | -.20 | 1 | 5 |
| **IT 9** | 3.66 | .83 | -.28 | -.10 | 1 | 5 |
| **IT 10** | 4.11 | .91 | -.80 | .05 | 1 | 5 |
| **IT 11** | 2.85 | .87 | .14 | -.01 | 1 | 5 |
| **IT 12** | 2.64 | .88 | -.00 | -.23 | 1 | 5 |
| **IT 13** | 2.25 | 1.23 | .65 | -.67 | 1 | 5 |
| **IT 14** | 3.62 | .84 | -.34 | -.01 | 1 | 5 |
| **IT 15** | 4.04 | .77 | -.64 | .67 | 1 | 5 |
| **IT 16** | 1.78 | .97 | 1.25 | 1.06 | 1 | 5 |
| **IT 17** | 2.92 | 1.02 | -.01 | -.52 | 1 | 5 |
| **IT 18** | 3.55 | .90 | -.20 | -.53 | 1 | 5 |
| **IT 19** | 3.79 | .88 | -.56 | .25 | 1 | 5 |
| **IT 20** | 3.11 | 1.08 | -.03 | -.65 | 1 | 5 |
| **IT 21** | 3.25 | .88 | -.12 | -.38 | 1 | 5 |
| **IT 22** | 3.72 | .85 | -.56 | .24 | 1 | 5 |
| **IT 23** | 2.46 | 1.19 | .44 | -.77 | 1 | 5 |
| **IT 24** | 4.13 | .79 | -.57 | -.20 | 1 | 5 |
| **IT 25** | 3.44 | .94 | -.31 | -.20 | 1 | 5 |
| **IT 26** | 3.78 | .78 | -.30 | -.00 | 1 | 5 |
| **IT 27** | 2.66 | 1.32 | .24 | -1.10 | 1 | 5 |
| **IT 28** | 3.58 | .82 | -.39 | .06 | 1 | 5 |
| **IT 29** | 3.21 | 1.06 | .06 | -.76 | 1 | 5 |
| **IT 30** | 3.31 | .94 | -.26 | -.22 | 1 | 5 |
| **IT 31** | 3.74 | .89 | -.61 | -.50 | 1 | 5 |
| **IT 32** | 3.49 | .77 | -.36 | .07 | 1 | 5 |
| **IT 33** | 3.90 | .87 | -.59 | .07 | 1 | 5 |
| **IT 34** | 3.49 | .83 | -.25 | .01 | 1 | 5 |
| **IT 35** | 3.95 | .87 | -.68 | .40 | 1 | 5 |
| **IT 36** | 3.77 | .88 | -.37 | -.29 | 1 | 5 |
| **IT 37** | 3.11 | .97 | -.06 | -.38 | 1 | 5 |
| **IT 38** | 3.55 | .84 | -.31 | -.09 | 1 | 5 |

*Note.* *N* = 587, *M* = mean scores, *SD* = standard deviations, *SK* = skewness, *Ku* = kurtosis, *Min* = minimum, *Max* = maximum.

**S2 Table B.** *Factor Loadings, inter-item correlations and internal consistency of the 30-item solution of the Multidimensional Will Scale (MWS).*

| ***Strong Will*** (α=.78 [.76‒.81], ω = .79 [.76‒.81]) | | | | |
| --- | --- | --- | --- | --- |
| **Factor Loadings** | | **Item-total**  **correlations** | **Cronbach’s α**  **(if item dropped)** | **McDonald’d ω**  **(if item dropped)** |
| **IT17** | .59 | .47 | .76 | .77 |
| **IT20** | .47 | .38 | .78 | .78 |
| **IT22** | .51 | .45 | .77 | .77 |
| **IT25** | .68 | .57 | .75 | .75 |
| **IT28** | .39 | .39 | .77 | .78 |
| **IT30** | .61 | .52 | .76 | .76 |
| **IT32** | .74 | .60 | .75 | .75 |
| **IT33** | .66 | .56 | .75 | .79 |
| **IT37** | .42 | .33 | .78 | .78 |
| ***Skillful Will*** (α=.66[.61‒.70], ω = .66 [.62‒.71]) | | | | |
| **Factor Loadings** | | **Item-total**  **correlations** | **Cronbach’s α**  **(if item dropped)** | **McDonald’d ω**  **(if item dropped)** |
| **IT2** | .66 | .44 | .60 | .60 |
| **IT9** | .32 | .37 | .63 | .64 |
| **IT35** | .69 | .51 | .56 | .56 |
| **IT36** | .54 | .44 | .60 | .60 |
| **IT38** | .35 | .32 | .65 | .66 |
| ***Good Will toward Self*** (α=.71[.67‒.74] , ω = .71 [.67‒.75]) | | | | |
| **Factor Loadings** | | **Item-total**  **correlations** | **Cronbach’s α**  **(if item dropped)** | **McDonald’d ω**  **(if item dropped)** |
| **IT3** | .52 | .45 | .66 | .67 |
| **IT6** | .62 | .47 | .65 | .66 |
| **IT10** | .42 | .38 | .69 | .71 |
| **IT14** | .83 | .55 | .62 | .62 |
| **IT24** | .56 | .48 | .65 | .66 |
| ***Good Will toward Others*** (α=.76 [.73‒.79], ω = .76 [.73‒.79]) | | | | |
| **Factor Loadings** | | **Item-totalItem**  **correlations** | **Cronbach’s α**  **(if item dropped)** | **McDonald’d ω**  **(if item dropped)** |
| **IT1** | .36 | .34 | .75 | .76 |
| **IT5** | .72 | .55 | .72 | .72 |
| **IT8** | .37 | .31 | .76 | .76 |
| **IT11** | .45 | .33 | .76 | .76 |
| **IT15** | .61 | .52 | .72 | .73 |
| **IT19** | .47 | .48 | .73 | .74 |
| **IT26** | .81 | .66 | .70 | .70 |
| **IT34** | .69 | .49 | .73 | .73 |
| ***Transpersonal Will*** (α=.83[.80‒.85], ω = .83[.80‒.85]) | | | | |
| **Factor Loadings** | | **Item-total**  **correlations** | **Cronbach’s α**  **(if item dropped)** | **McDonald’d ω**  **(if item dropped) *** |
| **IT4** | .81 | .68 | .76 | - |
| **IT13** | .82 | .67 | .77 | - |
| **IT23** | .84 | .70 | .74 | - |

*Note.* *Omega if item dropped requires at least 4 items to be computed.

**S3 Table C.** *Factor Loadings, inter-item correlations, and internal consistency of the 23-item Multidimensional Will Scale (MWS).*

| ***Strong Will*** (α= .78 [CI: .75 - .80]; ω= .78 [CI: .75 - .81]) | | | | |
| --- | --- | --- | --- | --- |
| **Factor Loadings** | | **Item-total**  **correlations** | **Cronbach’s α**  **(if item dropped)** | **McDonald’d ω**  **(if item dropped)** |
| **IT17** | .54 | .43 | .77 | .77 |
| **IT22** | .51 | .44 | .76 | .76 |
| **IT25** | .70 | .57 | .73 | .73 |
| **IT30** | .64 | .55 | .74 | .74 |
| **IT32** | .72 | .57 | .73 | .74 |
| **IT33** | .71 | .60 | .73 | .73 |
| ***killful Will*** (α= .65 [CI: .60 - 69]; ω= .66 [CI: .61 - .70]) | | | | |
| **Factor Loadings** | | **Item-total**  **correlations** | **Cronbach’s α**  **(if item dropped)** | **McDonald’d ω**  **(if item dropped)** |
| **IT2** | .64 | .42 | .59 | .60 |
| **IT9** | .33 | .36 | .63 | .65 |
| **IT35** | .71 | .52 | .51 | .52 |
| **IT36** | .58 | .43 | .58 | .61 |
| ***Good Will toward Self*** (α= .71 [CI: .67 - .74]; ω= .71 [CI: .67 - .75]) | | | | |
| **Factor Loadings** | | **Item-total**  **correlations** | **Cronbach’s α**  **(if item dropped)** | **McDonald’d ω**  **(if item dropped)** |
| **IT3** | .51 | .45 | .66 | .67 |
| **IT6** | .62 | .47 | .65 | .66 |
| **IT10** | .43 | .38 | .69 | .71 |
| **IT14** | .82 | .55 | .62 | .62 |
| **IT24** | .55 | .48 | .65 | .66 |
| ***Good Will toward Others*** (α= .76 [CI: .73 - .79]; ω=. 77 [CI: .74 - .80]) | | | | |
| **Factor Loadings** | | **Item-total**  **correlations** | **Cronbach’s α**  **(if item dropped)** | **McDonald’d ω**  **(if item dropped)** |
| **IT5** | .72 | .54 | .72 | .72 |
| **IT15** | .65 | .54 | .72 | .72 |
| **IT19** | .45 | .46 | .75 | .75 |
| **IT26** | .84 | .66 | .68 | .68 |
| **IT34** | .66 | .47 | .74 | .75 |
| ***Transpersonal Will*** (α= .83 [CI: .80 - .85]; ω= .83 [CI: .80 - .85]) | | | | |
| **Factor Loadings** | | **Item-total**  **correlations** | **Cronbach’s α**  **(if item dropped)** | **McDonald’d ω**  **(if item dropped)*** |
| **IT4** | .82 | .68 | .76 | *-* |
| **IT13** | .81 | .67 | .77 | - |
| **IT23** | .85 | .70 | .74 | - |

*Note.* *Omega if item dropped requires at least 4 items to be computed.
