# Supplementary material for: Measuring Strong, Skillful, Good and Transpersonal Will: The development of the Multidimensional Will Scale
Source: PLoS One. 2024 Jul 11;19(7):e0305477. doi: 10.1371/journal.pone.0305477 (PMC11239019; doi:10.1371/journal.pone.0305477)
Supplement: S1 Appendix — (DOCX) [file pone.0305477.s002.docx]

**Appendix 1:** The initial 38 items of the Multidimensional Will Scale (MWS).

1. When I have to make a choice, I consider understanding other people's point of view.

2. I try to envision the consequences of my decisions and choices.

3. I am committed to dedicating space in my life to what makes me feel good.

4. I feel the need to align my actions and choices with transcendent and spiritual values.

5. My first intention is to act in a way that does not harm anyone.

6. I neglect my body and my health.

7. I would like to transcend my human limitedness through union with someone/something bigger and higher.

8. I try to act with respect for the environment.

9. I patiently look for the best way to do things.

10. Doing something good for me makes me feel guilty.

11. I prefer to cooperate with others even if it slows me down in achieving my goals.

12. I give up what I like even though I know it hurts me.

13. My altruistic actions are inspired and supported also by my spiritual dimension.

14. I am committed to protecting and caring for myself.

15. When my decisions can be negative to others, I think about them carefully.

16. I perceive a contrast between my will and a "greater will" of a spiritual nature.

17. I have a hard time choosing and coming to a decision.

18. I devote time and energy to maturing and improving my personality.

19. When I make a choice, I feel responsible for the consequences of this choice on others.

20. I enjoy leading others and taking on roles of responsibility.

21. I am committed to abandoning my bad habits.

22. I recognize and use my inner qualities that can be useful to me in the pursuit of a goal.

23. In my choices I take into account profound and spiritual values.

24. Doing something good for myself makes me feel happy.

25. Obstacles motivate me to do more.

26. When I act, I keep in mind the welfare of others.

27. I recognize that there is a will superior to individual will whatever you call it: fate, Providence, etc.

28. I know how to realistically evaluate the chances of achieving my goals.

29. If I don't feel like doing something I can say "no" to people who ask me to do it.

30. When faced with difficulties, I feel discouraged.

31. I feel the need for my actions and choices to be consistent with the meaning I recognize in my life.

32. I am good at finding solutions to overcome obstacles.

33. I am determined to achieve the goals I set for myself.

34. I am willing to give up a part of my time and my plans to help others.

35. Before doing something important I think about its consequences over time.

36. I am careful to act in a way that protects myself from harmful consequences.

37. I am swayed by other people's opinions.

38. When I have to do something, I think about the easiest and most practical way of doing it
