# Supplementary material for: Measuring Strong, Skillful, Good and Transpersonal Will: The development of the Multidimensional Will Scale
Source: PLoS One. 2024 Jul 11;19(7):e0305477. doi: 10.1371/journal.pone.0305477 (PMC11239019; doi:10.1371/journal.pone.0305477)
Supplement: S2 Appendix — (DOCX) [file pone.0305477.s003.docx]

**Appendix 2:** The final 19 items of the Multidimensional Will Scale (MWS).

1. I try to envision the consequences of my decisions and choices.

2. I am committed to dedicating space in my life to what makes me feel good.

3. I feel the need to align my actions and choices with transcendent and spiritual values.

4. My first intention is to act in a way that does not harm anyone.

5. I neglect my body and my health.

6. My altruistic actions are inspired and supported also by my spiritual dimension.

7. I am committed to protecting and caring for myself.

8. When my decisions can be negative to others, I think about them carefully.

9. When I make a choice, I feel responsible for the consequences of this choice on others.

10. In my choices I take into account profound and spiritual values.

11. I have a hard time choosing and coming to a decision.

12. Doing something good for myself makes me feel happy.

13. Obstacles motivate me to do more.

14. When I act, I keep in mind the welfare of others.

15. When faced with difficulties, I feel discouraged.

16. I am good at finding solutions to overcome obstacles.

17. I am determined to achieve the goals I set for myself.

18. Before doing something important I think about its consequences over time.

19. I am careful to act in a way that protects myself from harmful consequences.
