# Supplementary material for: Measuring Strong, Skillful, Good and Transpersonal Will: The development of the Multidimensional Will Scale
Source: PLoS One. 2024 Jul 11;19(7):e0305477. doi: 10.1371/journal.pone.0305477 (PMC11239019; doi:10.1371/journal.pone.0305477)
Supplement: Attachment — Submitted filename: Review report.docx [file pone.0305477.s007.docx]

Thank you for giving me this opportunity to review the manuscript (No: PONE-D-23-06766).

This study aimed to develop and validate a Multidimensional Will Scale (MWS). I applaud the authors’ efforts to identify the dimensions of will according to Assagioli’s model and develop the preliminary 38-item version of the MWS in three phases, examine the factor structure of the 38-item MWS using exploratory factor analysis (Study 1), and identify the best-fit model using confirmatory factor analysis (Study 2).

Major issues:

1. The item selection procedure and the cut-off values used to remove items are not indicated in lines 258 to 260. As a result, the decision to remove items 7, 16, and 27 because of the skewness and/or kurtosis values does not seem justifiable. The values were below 2.00 and are considered acceptable to some researchers. The authors are urged to explain the reason for using skewness and kurtosis, rather than factor loading, to determine the items that shall be removed. Finally, the kurtosis value of item 27 reported in line 276 is different from the value reported in S1 Table.
2. Lines 287 to 286, the number of factors to retain in the parallel analysis is not decided by the eigenvalue > 1 criterion, but by the number of eigenvalues (generated from the dataset) that are larger than the corresponding random eigenvalues.
3. Lines 291 to 292, “the analyses for a 31-item solution, with the number of factors fixed at five, were repeated.” The writing does not indicate whether the researchers delete one of the four items (i.e., items 18, 21, 29, 31) each time and then rerun the EFA. In addition, it is not justifiable to fix the number of factors. Note that the number of factors is likely to change when an item is removed.
4. Line 293, “IT12 (r=.31) has been eliminated as statistically non-significant.” It is not clear if the reported r-value refers to factor loading. Moreover, the authors did not mention that statistical significance is one of the criteria for determining item removal.
5. Lines 301 to 302, “The content analysis led to moving these two items from the Skillful to the Strong Will dimension” The writing seems to indicate that the authors think that it is acceptable to keep items 22 and 32 to Strong Will dimension. The items, however, were evaluated by experts and recommended to be loaded on Skillful Will dimension. The inconsistent decisions require further explanation.
6. Lines 319 to 320 and Lines 342 to 343, items 9 and 10 with a factor loading below 0.40 were not removed to maintain the Cronbach alpha. This practice, again, does not seem justifiable.
7. Lines 329, Is the result of the scree test consistent with the parallel analysis result? It is confusing why the authors referred to the scree test result.
8. Lines 506 to 513, items 10, 9, 22, and 34 were removed and the retained items were submitted to CFA. The CFA results shall be interpreted as exploratory rather than confirmatory. A new set of data shall be collected to confirm the revised structure/model.

Minor issue

1. The authors use the term will and volition in the literature review. It is important to clarify if the two terminologies are conceptually equivalent.
2. Lines 246 to 249, explain the reason to run two EFAs using PC and FA respectively.

I did not review the Discussion because the abovementioned major issues lead me to doubt the appropriateness of the selected items.
