# Supplementary material for: Measuring Strong, Skillful, Good and Transpersonal Will: The development of the Multidimensional Will Scale
Source: PLoS One. 2024 Jul 11;19(7):e0305477. doi: 10.1371/journal.pone.0305477 (PMC11239019; doi:10.1371/journal.pone.0305477)
Supplement: Attachment — Submitted filename: Response to Reviewers.docx [file pone.0305477.s008.docx]

**Title:** Assessment of will from psychodynamic theory: The development of the Multidimensional Will Scale

**Journal:** PLOS ONE

Dear Silva Ibrahimi,

Thank you for your letter dated July 31, 2023. We are sincerely grateful that you are willing to consider a revised draft of our manuscript for potential publication in the PLOS One.

We express our gratitude to the Editor and the Reviewer for their insightful comments and suggestions. We have diligently made revisions to the manuscript in accordance with their feedback. Attached, you will find a copy of the revised manuscript, which includes tracked changes, along with an unmarked version. Additionally, we have provided detailed point-by-point responses to the reviewer's comments. In the revised manuscript, all modifications made to the previous version are meticulously tracked.

We would also like to take this opportunity to thank you for helping us to improve the quality of our manuscript.

Kind regards,

Andrea Bonacchi

---

**Editor:**

**Comment 1.** Please ensure that your manuscript meets PLOS ONE's style requirements, including those for file naming.

**Response.** *We thank the Editor for guiding us on how to improve our manuscript to make it compliant with the PlosOne guidelines. We have made the necessary changes.*

**Comment 2.** Please provide additional details regarding ethical approval in the body of your manuscript. In the Methods section, please ensure that you have specified the name of the IRB/ethics committee that approved your study.

**Response.** *We thank the Editor for pointing out the lack of this information. We have added more details in the Method section of Study 1 (lines 227-228) and Study 2 (lines 418-419). The ethical committee's approval has been translated and attached during the submission process, and all the necessary information typically found in scientific publications has been reported in the method section.*

**Comment 3.** Please include your full ethics statement in the ‘Methods’ section of your manuscript file. In your statement, please include the full name of the IRB or ethics committee who approved or waived your study, as well as whether or not you obtained informed written or verbal consent. If consent was waived for your study, please include this information in your statement as well.

**Response.** *We thank the Editor for pointing out the lack of this information. We have already partially addressed this in response to comment 2 and have also added more details in the Method section of Study 1 (lines 229-230) and Study 2 (line 423).*

**Reviewer #1:**

**Comment 1.** The item selection procedure and the cut-off values used to remove items are not indicated in lines 258 to 260. As a result, the decision to remove items 7, 16, and 27 because of the skewness and/or kurtosis values does not seem justifiable. The values were below 2.00 and are considered acceptable to some researchers. The authors are urged to explain the reason for using skewness and kurtosis, rather than factor loading, to determine the items that shall be removed. Finally, the kurtosis value of item 27 reported in line 276 is different from the value reported in S1 Table.

**Response.** *We thank the reviewer for pointing out the need to include the criteria that guided us in selecting the most suitable items for the scale. We have added the necessary information in lines 245-251. We have also corrected the kurtosis value for item 27 and apologize for the typo.*

**Comment 2.** Lines 287 to 286, the number of factors to retain in the parallel analysis is not decided by the eigenvalue > 1 criterion, but by the number of eigenvalues (generated from the dataset) that are larger than the corresponding random eigenvalues.

**Response.** *We would like to thank the reviewer for bringing this point to our attention. We have rewritten the sections on the analyses performed (lines 255-269) to better illustrating the employed procedure. Please, see also the response to Comment 10.*

**Comment 3.** Lines 291 to 292, “the analyses for a 31-item solution, with the number of factors fixed at five, were repeated.” The writing does not indicate whether the researchers delete one of the four items (i.e., items 18, 21, 29, 31) each time and then rerun the EFA. In addition, it is not justifiable to fix the number of factors. Note that the number of factors is likely to change when an item is removed.

**Response**. *We thank the reviewer for the comment.* *Determining the appropriate number of factors to retain in exploratory factor analysis (EFA) is a topic frequently discussed among researchers in the field of psychometrics. While certain guidelines indicate that there is no single correct method, the decision on which factors to retain and the criteria used are contingent upon the specific goals underlying the EFA. As such, fixing the number of factors in EFA is justified when a well-established theoretical framework initially hypothesizes a specific number of dimensions, as is the case in MWS scale development where five distinct dimensions were theorized. By adhering to this theoretical foundation and fixing the number of factors to five, the analysis maintains consistency with the initial hypotheses, enhances interpretability, and minimizes the risk of overfitting while providing a data-driven rationale based on the combination of theoretical guidance and initial empirical support. This approach aligns with research aims, simplifies factor interpretation, and sets the stage for potential confirmatory analyses such as confirmatory factor analysis (CFA) to further validate the chosen factor structure against the data. We have also specified that the four items with loadings below .30 were eliminated before proceeding with further analyses (lines 313-314 and lines 317-318).*

**Comment 4**. Line 293, “IT12 (r=.31) has been eliminated as statistically non-significant.” It is not clear if the reported r-value refers to factor loading. Moreover, the authors did not mention that statistical significance is one of the criteria for determining item removal.

**Response.** *We thank the reviewer for providing guidance on enhancing the clarity and precision of our manuscript. Consequently, we have implemented revisions in the text (lines 311-312) to address the concerns highlighted.*

**Comment 5.** Lines 301 to 302, “The content analysis led to moving these two items from the Skillful to the Strong Will dimension” The writing seems to indicate that the authors think that it is acceptable to keep items 22 and 32 to Strong Will dimension. The items, however, were evaluated by experts and recommended to be loaded on Skillful Will dimension. The inconsistent decisions require further explanation.

**Response.** *We extend our gratitude to the reviewer for providing insights on enhancing the clarity of our explanations concerning the repositioning of the two items between dimensions. A clearer sentence can be found within lines 320-323 and within the Discussion section of Study 1 (lines 376-382).*

**Comment 6.** Lines 319 to 320 and Lines 342 to 343, items 9 and 10 with a factor loading below 0.40 were not removed to maintain the Cronbach alpha. This practice, again, does not seem justifiable.

**Response.** *We thank the reviewer for raising this question. We have clarified in the text that these values refer to Item-total correlation which is just one of the reliability measures being considered and not to factor loadings (lines 328-330 and line 340). Given the limited number of items per dimension, which could have impacted the psychometric properties of each dimension, it was preferable to retain the items whose item-total correlation was only slightly below the threshold, specifically IT9 (r=.37) and IT10 (r=.38). These items, despite their item-total correlation values, contributed significantly to the reliability of the dimension. Furthermore, in the 23-item version, where reliability measures were repeated, only IT10 retained the same value (r=.38). Following the same rationale, it was preserved within the scale.*

**Comment 7.** Lines 329, Is the result of the scree test consistent with the parallel analysis result? It is confusing why the authors referred to the scree test result.

**Response.** *We express our appreciation to the reviewer for proposing the inclusion of significant explanations concerning the decision to reference the results of a scree test. As outlined in lines 307-308 and 349-350, the Scree test confirmed the 5-factor solution, aligning with the outcomes of prior analyses. Regarding the choice to consider the scree test analysis, we adhered to practices commonly recommended in the literature, including the use of Kaiser’s criterion and the Scree test for determining the appropriate number of factors to retain. Indeed, especially when theoretical models underpin analyses, combining these two methods ensures an optimal approach to factor selection (Yong & Pearce, 2013). In addition, in response to reviewer’s comment 2, we have rewritten and provided a clearer explanation of the analysis strategy, where the decision to use the scree-test is also justified (see Analysis section).*

**Comment 8.** Lines 506 to 513, items 10, 9, 22, and 34 were removed and the retained items were submitted to CFA. The CFA results shall be interpreted as exploratory rather than confirmatory. A new set of data shall be collected to confirm the revised structure/model.

**Response.** *We appreciate the reviewer for providing the opportunity to clarify this aspect further. In Study 1, the initial scale underwent a series of statistical analyses, including EFA, to refine its psychometric properties, encompassing the removal of inadequate items. In Study 2, we already had a reduced scale with 5 defined dimensions. Thus, Study 2, sufficed to confirm its factorial structure, leading to the execution of confirmatory factor analysis (CFA). However, the main objective of Study 2, for which data were newly collected, was the further examination and enhancement of the psychometric properties of the MWS. Therefore, based on the conducted analyses, additional items were removed. Two data collections were conducted, resulting in two studies that led to the development of a 19-item version of the MWS and confirmed its strong psychometric properties, as well as adherence to the theoretical model upon which we initially based our research. Future studies will aim to further confirm the current findings.*

**Comment 9.** The authors use the term will and volition in the literature review. It is important to clarify if the two terminologies are conceptually equivalent.

**Response.** *We thank the reviewer for suggesting the clarification of the two terms. The necessary clarifications have been made in lines 99-102.*

**Comment 10.** Lines 246 to 249, explain the reason to run two EFAs using PC and FA respectively.

**Response.** *We apologize for the previous misleading analysis description. Now we provide a detailed explanation in lines 255-269. Specifically, we performed a parallel analysis based on the principal component (PC) to reduce the number of variables by maximally preserving information from the original dataset (Watkins, 2018) and the EFA (based on the principal axis factoring extraction method) that must be preferred to better represent the underlying latent construct (Cudeck 2000).*
